# Increased Long-Term Mortality among Black CABG Patients Receiving Preoperative Inotropic Agents

**DOI:** 10.3390/ijerph120707478

**Published:** 2015-07-06

**Authors:** Jimmy T. Efird, William F. Griffin, Daniel F. Sarpong, Stephen W. Davies, Iulia Vann, Nathaniel T. Koutlas, Ethan J. Anderson, Patricia B. Crane, Hope Landrine, Linda Kindell, Zahra J. Iqbal, T. Bruce Ferguson, W. Randolph Chitwood, Alan P. Kypson

**Affiliations:** 1 Department of Cardiovascular Sciences, Brody School of Medicine, East Carolina Heart Institute, East Carolina University, Greenville, NC 27834, USA; E-Mails: griffinw11@students.ecu.edu (W.F.G); koutlasnate@gmail.com (N.K.); kindelll@ecu.edu (L.K.); iqbalz@ecu.edu (Z.J.I.); fergusont@ecu.edu (T.B.F.); chitwoodw@ecu.edu (W.R.C.); kypsona@ecu.edu (A.P.K.); 2 Center for Health Disparities, Brody School of Medicine, East Carolina University, Greenville, NC 27834, USA; E-Mail: landrineh@ecu.edu; 3 Department of Public Health, Brody School of Medicine, East Carolina University, Greenville, NC 27834, USA; E-Mail: vanni13@students.ecu.edu; 4 Center for Minority Health and Health Disparities Research and Education, Xavier University of Louisiana, New Orleans, LA 70125, USA; E-Mail: dsarpong@xula.edu; 5 Department of General Surgery, University of Virginia School of Medicine, Charlottesville, VA 22908, USA; E-Mail: SD2WF@hscmail.mcc.virginia.edu; 6 Department of Pharmacology and Toxicology, Brody School of Medicine, East Carolina University, Greenville, NC 27834, USA; E-Mail: andersonet@ecu.edu; 7 The College of Nursing, East Carolina University, Greenville, NC 27834, USA; E-Mail: cranep14@ecu.edu

**Keywords:** inotropes, cardiac surgery, mortality, disparities, heart disease, heart failure

## Abstract

The aim of this study was to examine racial differences in long-term mortality after coronary artery bypass grafting (CABG), stratified by preoperative use of inotropic agents. Black and white patients who required preoperative inotropic support prior to undergoing CABG procedures between 1992 and 2011 were compared. Mortality probabilities were computed using the Kaplan-Meier product-limit method. Hazard ratios (HR) and 95% confidence intervals (CI) were computed using a Cox regression model. A total of 15,765 patients underwent CABG, of whom 211 received preoperative inotropic agents within 48 hours of surgery. Long-term mortality differed by race (black *versus* white) among preoperative inotropic category (inotropes: adjusted HR = 1.6, 95% CI = 1.009–2.4; no inotropes: adjusted HR = 1.15, 95% CI = 1.08–1.2; *P*_interaction_ < 0.0001). Our study identified an independent preoperative risk-factor for long-term mortality among blacks receiving CABG. This outcome provides information that may be useful for surgeons, primary care providers, and their patients.

## 1. Introduction

Inotropic agents are used for the treatment of hypotension and hypoperfusion syndrome in the setting of acute decompensated heart failure or cardiogenic shock after acute myocardial infarction (MI) [1]. While inotropes have been shown to improve hemodynamics, potential complications associated with their use include myocardial ischemia secondary to increased oxygen demand and stunning of myocardium in ischemic heart disease. The perioperative use of inotropic agents has been associated with increased morbidity and mortality in patients undergoing open heart surgery [2].

Differences between ethnic groups in responsiveness to several classes of cardiovascular drugs (e.g., warfarin, β-blockers, angiotensin-converting-enzyme inhibitors, angiotensin receptor blockers and isosorbide-hydralazine) have been well documented [3]. Although short-term outcomes of inotrope use among black patients have been examined in the literature, information is generally lacking on the long-term effect of their preoperative use in this population [4]. Because ethnicity often is an important factor in guiding drug therapy and follow-up care, we sought to explore the differential long-term mortality of preoperative inotrope use by race among patients undergoing coronary artery bypass graft (CABG) surgery. We hypothesize that long-term mortality would be increased among black patients who had ischemic cardiomyopathy and received preoperative inotropes.

## 2. Methods

### 2.1. Study Design

This was a retrospective cohort study of 15,765 patients undergoing first-time CABG at the East Carolina Heart Institute from 1992 to 2011. Demographic data, medication use, comorbid conditions, CAD severity, and surgical data were collected at the time of surgery. Less than 1% of patients were races other than black or white and were excluded to minimize the potential for residual confounding. Racial identity was self-reported. The study was approved by the Institutional Review Board at the Brody School of Medicine, East Carolina University (UMCIRB 12-002107). This was a natural history (non-experimental) analysis of an archival de-identified dataset, in consent with the Declaration of Helsinki. Details of the study database and methodology have been previously described and are briefly summarized below [5,6,7,8,9,10,11,12,13,14,15].

### 2.2. Definitions

In this study, patients were categorized into the inotrope group if within the 48 hour window prior to surgery they received an inotropic agent (e.g., β-adrenergic agonist: dobutamine, dopamine, epinephrine, norepinephrine; phosphodiesterase-III inhibitor: amrinone, enoximone, milirinone, theophylline). The patients could have been in acute cardiogenic shock secondary to ischemic heart disease or acute decompensated heart failure. Heart failure was defined as having a preoperative diagnosis according to standard Society of Thoracic Surgeons (STS) criteria. This included physician documentation or confirmatory medical reports (hospital admission notes, chest radiographs, consultations, physical exam, medication administration records, outpatient records, and radiology reports). Stable or asymptomatic compensated failure or patients whose symptoms improved after medical therapy were not included in this definition. Patients with a low ejection fraction without clinical symptoms (edema, rales, paroxysmal nocturnal dyspnea) also were not defined as having heart failure. Adjudication review was used to rule out non-definitive heart failure diagnoses. Only definitive cases were included in this study. CAD severity was defined by the number of diseased vessels (1, 2, or 3) with at least 50% stenosis and confirmation by angiography before surgery. Long-term mortality was defined as any cause of death occurring postoperatively.

### 2.3. Setting

The East Carolina Heart Institute is a large (>120 beds) cardiovascular hospital located in rural North Carolina, a region with a large black population. The institute is the largest stand-alone, tertiary referral hospital focusing on diagnoses and treatment of cardiovascular disease in the state of North Carolina. Cardiovascular disease is a leading cause of death in North Carolina with an unequal burden occurring in eastern North Carolina [16]. The majority of patients treated at the East Carolina Heart Institute live and remain within a 150-mile radius of the medical center.

### 2.4. Data Collection and Follow-Up

Study data were extracted from the Society of Thoracic Surgeons (STS) Adult Cardiac Surgery Database and the electronic medical record at the Brody School of Medicine. Data quality and cross-field validation are routinely performed by the Center for Epidemiology and Outcomes Research at the East Carolina Heart Institute. Electronic medical records were first introduced at the Brody School of Medicine in 1997. Patient information prior to 1997 was retrospectively scanned into the electronic medical record, with complete conversion beginning in 1992. Data on intra/postoperative inotrope use was only collected from 1992–2007 (n = 13,751). Local and regional health care providers were networked under a single electronic medical record system in 2005 which allowed for efficient follow-up. Multiple logic comparisons are applied to the data to reduce mismatching of information across clinics and follow-up visits. Our cardiovascular surgical database is linked to the electronic medical record through a unique patient medical record number.

The catchment area for the East Carolina Heart Institute is relatively stable. In most cases, deaths are documented through the regional medical center electronic medical record system, follow-up letters/phone calls (primary care providers, patients), death certificates from county registrars, and/or obituary notices. The National Death Index was used to validate death information in our electronic medical record and complete missing data. Less than 5% of deaths in our database failed to correctly match with the National Death Index.

### 2.5. Statistical Analysis

Categorical variables were reported as frequency and percentage while continuous variables were reported as median and interquartile range. Follow-up time was computed from the date of surgery to the date of death or censoring. The Kaplan-Meier product-limit method was used to estimate mortality/survival probabilities, with differences between groups tested using the log-rank procedure. Cox proportional hazard regression was used to compute hazard ratios (HR) and 95% confidence intervals (CI) for long-term mortality. The initial multivariable models included variables that have been previously reported to be associated with cardiovascular-related mortality, regardless of their statistical significance in our dataset. These included race, age, sex, hypertension, CAD severity, heart failure, and prior stroke [9]. The *post-hoc* addition of other variables into the model was performed in a pairwise manner. The test statistic of Grambsch and Therneau was used to validate the proportional hazards assumption [17]. Statistical significance for categorical variables was tested using the Fisher’s Exact Test and the Deuchler-Wilcoxon method for continuous variables. P_Trend_ for increasing or decreasing HRs across levels of continuous variables was computed using a likelihood ratio test. An iterative expectation-maximization (EM) algorithm was applied to impute missing values [18]. Similarly, the interaction between preoperative inotrope use and race was tested using a likelihood ratio test. The method of Holly and Whittemore was used for rounding [19]. Statistical significance was defined as *p* < 0.05. SAS Version 9.4 (Cary, NC, USA) was used for this analysis.

## 3. Results and Discussion

### 3.1. Results

A total of 15,765 patients received CABG during the study period from 1992 to 2011. The median follow-up time in years was 7.8 with an interquartile range (IQR) of 8.3 (blacks: median = 6.4, IQR = 7.8; whites: 8.0, IQR = 8.3). Black and white patients differed with respect to BMI (median), dialysis (%) and peripheral arterial disease (PAD) (%) in both the preoperative inotrope and non-inotrope group (Table 1). No statistically significant differences were observed for preoperative medications between black and white patients on preoperative inotropes (Table 2). Similarly, blacks and whites on preoperative inotropes did not differ with respect to postoperative complications (Table 3).

Among back patients, 56% received an intra/postoperative inotropic agent *versus* 49% for white patients (Fisher’s exact *p* < 0.0001, not shown in tables). However, few patients received both a preoperative and intra/postoperative inotropic agent (1.2%).

**Figure 1:**
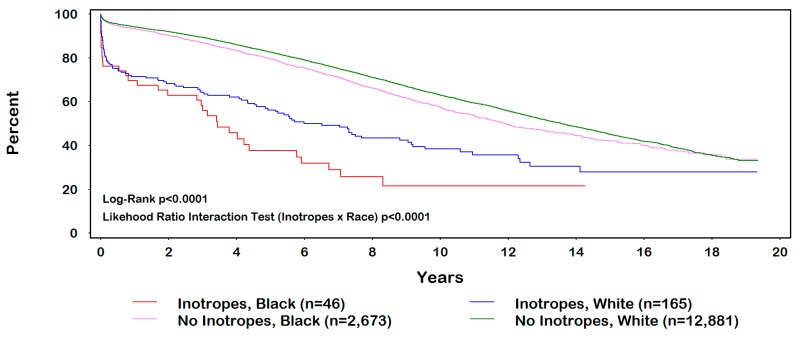
Inotrope use by race.

Long-term mortality differed by race (black *vs.* white) among preoperative inotropic category (inotropes: adjusted HR = 1.6, 95% CI = 1.009–2.4; no inotropes: adjusted HR = 1.15, 95% CI = 1.08–1.2; *P*_interaction_ < 0.0001) (See Table 4). Kaplan-Meier survival plot is shown in Figure 1. The multivariable results did not substantively change with the pairwise inclusion of other variables listed in (Table 1) and (Table 2). Because inotropes are used to treat heart failure and may have resulted in over-adjustment, our multivariable models were repeated excluding the variable heart failure, with no significant effects on our results. No critical departures from model fit where noted for the Cox regression analyses performed in our study.

**Table 1:** Patient characteristics (N = 15,765).

Characteristic	Inotropes		*p*-Value *	No Inotropes		*p*-Value *
	Black n (%)	White n (%)		Black n (%)	White n (%)	
Overall	46 (22)	165 (78)	–	2673 (17)	12881 (83)	–
Age (Years)						
Median (IQR)	68 (20)	66 (15)	0.49	62 (16)	65 (15)	<0.0001
Female	20 (43)	53 (32)	0.16	1131 (42)	3624 (28)	<0.0001
BMI (kg/m^2^) **^£^**						
Median (IQR)	29 (6.8)	27 (5.7)	0.019	29 (7.5)	28 (6.6)	<0.0001
Ejection fraction (%) **^£^**						
Median (IQR)	39 (20)	35 (25)	0.21	50 (20)	52 (20)	<0.0001
Isolated CABG	34 (74)	130 (79)	0.55	2345 (88)	11265 (87)	0.72
Surgery prior to Year 2001	16 (35)	80 (48)	0.13	1275 (48)	7503 (58)	<0.0001
CAD severity			0.36			0.046
1 Vessel	1 (2)	12 (7)		219 (8)	1215 (9)	
2 Vessel	12 (26)	51 (31)		702 (26)	3507 (27)	
3 Vessel	33 (72)	102 (62)		1752 (66)	8159 (63)	
Left main disease	9 (20)	43 (26)	0.44	528 (20)	2535 (20)	0.94
Recent smoker	11 (24)	54 (33)	0.28	680 (25)	3023 (23)	0.030
Hypertension	39 (85)	93 (56)	0.0005	2289 (86)	8985 (70)	<0.0001
Diabetes	28 (61)	54 (33)	0.0004	1256 (47)	4078 (32)	<0.0001
Dialysis	4 (9)	1 (1)	0.0085	166 (6)	104 (1)	<0.0001
Heart failure ^‡^	26 (57)	87 (53)	0.74	699 (26)	2310 (18)	<0.0001
Peripheral arterial disease	12 (26)	17 (10)	0.013	385 (14)	1511 (12)	0.0002
Prior MI	33 (72)	126 (76)	0.56	1141 (43)	4822 (37)	<0.0001
Prior stroke	5 (11)	13 (8)	0.55	302 (11)	975 (8)	<0.0001
Prior PCI	12 (26)	54 (33)	0.47	521 (19)	2463 (19)	0.67

* Fisher’s Exact (categorical) and Deuchler-Wilcoxon (continuous) test for comparing variables for Black *vs.* White patients within ionotropic group. **^£^** Estimates computed using the EM algorithm (n = 10 simulations, ^£^ imputational efficiency ≥ 99%). **^‡^** Stable or asymptomatic compensated. BMI = body mass index; CABG = coronary artery bypass graft; CAD = coronary artery disease; IQR = interquartile range; MI = myocardial infarction; PCI = percutaneous coronary intervention; SD = standard deviation.

**Table 2:** Preoperative medications.

Medications	Inotropes		*p*-Value *	No Inotropes		*p*-Value *
	Black n (%)	White n (%)		Black n (%)	White n (%)	
Aspirin	32 (70)	104 (63)	0.49	1800 (67)	8891 (69)	0.090
Lipid Lowering Agents	11 (24)	45 (27)	0.71	1186 (44)	5088 (40)	<0.0001
β-blockers	19 (41)	64 (39)	0.86	1631 (61)	6940 (54)	<0.0001
Anticoagulants	31 (67)	116 (70)	0.72	844 (32)	4026 (31)	0.75
Antiplatelet Agents	21 (46)	92 (56)	0.25	1198 (45)	6542 (51)	<0.0001
Calcium Channel Blockers	8 (17)	21 (13)	0.47	880 (33)	3857 (30)	0.0025
Diuretics	23 (50)	77 (47)	0.74	814 (30)	2824 (22)	<0.0001
ACE Inhibitors/ARBs	14 (36)	54 (33)	0.86	1095 (41)	3808 (30)	<0.0001
Digitalis	10 (22)	19 (12)	0.090	180 (7)	1003 (8)	0.065
Nitrates	19 (41)	76 (46)	0.62	356 (13)	1983 (15)	0.0062

* Fisher’s exact test comparing categorical variables for race within inotrope group.

**Table 3:** Postoperative complications.

Complications	Inotropes		*p*-Value *	No Inotropes		*p*-Value *
	Blacks n (%)	Whites n (%)		Blacks n (%)	Whites n (%)	
MI	0 (0)	1 (1)	1.0	5 (<1)	59 (<1)	0.046
Stroke	0 (0)	3 (2)	1.0	71 (3)	184 (1)	<0.0001
ARDS	3 (7)	15 (9)	0.77	19 (1)	119 (1)	0.31
Pneumonia	1 (2)	16 (10)	0.13	68 (3)	257 (2)	0.75
Gastrointestinal	2 (4)	18 (11)	0.26	103 (4)	307 (2)	<0.0001

* Fisher’s exact test comparing categorical variables for race within inotrope group. ARDS = acute respiratory distress syndrome; MI = myocardial infarction.

**Table 4:** Univariable and multivariable hazard ratios for mortality.

Characteristic ^†^	Inotropes		No Inotropes	
	Univariable	Multivariable	Univariable	Multivariable
	HR (95% CI)	HR (95% CI)	HR (95% CI)	HR (95% CI)
Black ^*^	1.5 (1.004–2.2)	1.6 (1.009–2.4)	1.15 (1.08–1.2)	1.15 (1.08–1.2)
Age in years (continuous)	1.04 (1.02–1.06)	1.04 (1.02–1.1)	1.057 (1.055–1.060)	1.054 (1.051–1.057
Female	1.2 (0.82–1.7)	0.99 (0.68–1.4)	1.13 (1.07–1.2)	0.93 (0.88–0.98)
Hypertension	1.1 (0.77–1.6)	0.81 (0.55–1.2)	1.26 (1.2–1.3)	1.1 1.07–1.2)
CAD severity				
1 Vessel	1.0 Referent	1.0 Referent	1.0 Referent	1.0 Referent
2 Vessel	1.1 (0.47–2.7)	0.92 (0.38–2.2)	1.2 (1.08–1.3)	1.2 (1.1–1.4)
3 Vessel	1.6 (0.72–3.4)	1.3 (0.57–3.1)	1.4 (1.3–1.6)	1.4 (1.3–1.5)
	*P*_trend_ = 0.048 ^§^	*P*_trend_ = 0.10 ^§^	*P*_trend_ < 0.0001 ^§^	*P*_trend_ < 0.0001 ^§^
Heart failure^‡^	1.3 (0.89–1.8)	1.1 (0.75–1.6)	2.2 (2.1–2.3)	1.8 (1.7–2.0)
Prior stroke	1.2 (0.65–2.3)	1.2 (0.62–2.2)	1.9 (1.8–2.1)	1.6 (1.5–1.8)

^*^ Multivariable *P*_interaction_ < 0.0001 (Likelihood ratio test, Inotropes x Race). **^§^** Likelihood ratio trend test. **^‡^** Stable or asymptomatic compensated. CAD = coronary artery disease; CI = confidence interval; HR = hazard ratio. **^†^** Referent groups were white, male, no hypertension, no heart failure and no prior stoke, respectively.

## 3.2. Discussion

In our study a statistically significant interaction was observed between preoperative inotrope use and race with respect to long-term mortality. To our knowledge this is the first study to examine long-term mortality among black CABG patients receiving preoperative inotropes. The use of preoperative inotropes was associated with increased risk of long-term mortality in blacks compared with whites. Possible explanations for this finding may include differences in heart failure etiology, drug metabolism and physiologic response to inotropes.

Racial differences in short-term mortality in patients with acute decompensated heart failure who received inotropic therapy was examined in the OPTIME-CHF (Outcomes of a Prospective Trial of Milrinone for Exacerbations of Chronic Heart Failure) study [4]. This prospective randomized trial reported statistically significant lower 60-day mortality (unadjusted analysis) among black patients who received milirinone, compared with whites. However, no difference in mortality was detected after the adjustment for baseline differences. Even though the OPTIME-CHF trial excluded patients who received inotropic therapy for cardiogenic shock and those with preserved systolic function, the adjusted results correspond closely to our findings over the same 60-day time period. The majority (74%) of blacks enrolled in the OPTIME-CHF trial had non-ischemic heart failure. In contrast, patients in our study were assumed to have cardiac ischemia secondary to underlying coronary artery disease requiring surgical bypass.

The OPTIME-CHF study also compared the use of inotropes in ischemic *versus* non-ischemic heart failure patients [20]. Those enrolled in the study with non-ischemic heart failure tolerated or even benefited from the use of inotropes in comparison with ischemic heart failure patients who experienced increased mortality and hospitalization rates. The results of the study suggest that a differential response to inotropes by race may be attributable to differing etiologies of heart failure.

While patients in our study were primarily treated for ischemic heart disease, an increased rate of diabetes and hypertension was observed among black patients, which is frequently associated with diabetic or hypertensive cardiomyopathy. Altered inotropic responses have been reported in diabetic cardiomyopathy and hypertensive-diabetic cardiomyopathy within the laboratory setting. Specifically, inotropes administered to diabetic and hypertensive rodents have higher sensitivity and effect on the myocardium which presumably leads to increased mortality [21]. Conceivably, similar effects might underlie the differential mortality outcome that is seen in black patients on inotropic agents. Another contributing factor, as noted in prior studies, may be that black patients receive life-saving cardioprotective therapies less often than whites [22]. While inotropic agents may improve mitochondrial function in noninfarcted myocardial tissue, free calcium in the cytosol can lead to activation of proteolytic enzymes, proapoptotic signal cascades, mitochondrial damage and eventually, necrosis and disrupted membranes [23].

Our findings are important to contrast with the African-American Heart Failure Trial (A-HeFT) [24] of combination isosorbide dinitrate and hydralazine in black patients. This trial showed that nitric oxide medications are more effective in black patients than whites. Isosorbide dinitrate functions by being converted to nitric oxide (NO), an active intermediate compound which activates the enzyme guanylate cyclase [25]. This stimulates the synthesis of cyclic guanosine 3’, 5’-monophosphate (cGMP) that in turn activates a series of protein kinase-dependent phosphorylations in the vascular smooth muscle cells and results in the dephosphorylation of the myosin light chain of the smooth muscle fiber. Subsequently, the release of calcium ions by the myosin light chain results in the relaxation of the smooth muscle cells and vasodilation. It is generally known that NO induces protein kinase G (PKG) which has a dual effect: (1) inhibition of inositol trisphosphate receptor (IP3) receptor resulting in calcium release from sarcoplasmic reticulum (SR) into cytoplasm, and (2) increased phospholamban phosphorylation, SERCA2a activity and calcium uptake into the SR [26,27,28]. Both effects will reduce the calcium release from SR into the cytoplasm and sequester more of the calcium inside the SR. Additionally, isosorbide dinitrate has been noted to have a weak positive inotropic effect on the myocardium [29]. Blacks with heart failure have been observed to exhibit greater NO reduction while whites have modestly reduced NO [30]. It can, therefore, be postulated that isosorbide dinitrate has a positive inotropic effect that is greater among black patients, although the vasodilatory effect of NO is likely to be the predominant factor. However, as reported in A-HeFT, blacks with heart failure had better outcomes (e.g., decreased mortality, improved quality of life at 6 months) when randomized to a combination of isosorbide dinitrate and hydralazine, which inhibits destruction of NO. While it is presumed that this effect is more pronounced in black patients, white patients were not enrolled in the above study as a referent group. Moreover, our database did not allow us to ascertain the prevalence of phosphodiesterase inhibitors as inotropic therapy in our patient cohort. This is a potentially important variable given that these agents are known to exhibit a NO-potentiating effect.

We cannot rule out that the differential long-term mortality observed in our study may be explained by factors unrelated to the preoperative use of inotropic agents. For example, residents of rural areas have limited access to medical care compared with urban regions and this effect may have been potentiated among black patients owing to historic racial barriers in the South [31,32]. Furthermore, blacks traditionally prioritize health to a lesser degree than whites, postpone seeing a doctor, often do not have a regular physician, and generally are less trusting of health care providers [33,34,35]. Other contributing factors may include suboptimal follow-up care, inappropriate specialist referral, non-adherence to treatment regimens, community segregation, differences in socioeconomic position and educational achievement, and other social determinants of health [36]. The use of inotropic agents in this population also might simply represent a surrogate marker of severity that is further compounded by the above mentioned factors. However, in previous studies of racial differences in long-term mortality following CABG surgery, blacks with chronic lung disease or renal failure requiring dialysis were observed to have equal or more favorable outcomes than whites [7,8].

Our study is strengthened by its large racially dichotomous population and systematic data collection. Nonetheless, various limitations should be noted when interpreting our results. Although we adjusted for patient demographics and several comorbidities associated with long-term mortality following CABG, residual confounding may still exist due to the non-randomized nature of this study and limited sample size within the inotrope group. Stable or asymptomatic compensated heart failure patients or patients whose symptoms improved after medical therapy were not categorized as being in heart failure by the STS definition and this may have further resulted in incomplete adjustment when added pairwise to our multivariable Cox regression model. Type, dose, duration and reason for inotrope use were not recorded in our STS database and this information frequently was incomplete or not available in retrospective scans of medical charts. Accordingly, we were unable to determine the differential clinical impact, for example, of being on dopamine for 3 h preoperatively *versus* 2 days of high dose epinephrine. The possibility also exists that some patients may have received low-dose inotropic agents for indications other than heart failure or cardiogenic shock after acute MI (e.g., decreased renal profusion). However, any bias resulting from including these cases in our analyses likely would have been toward the null.

The absence of individual and area-level socioeconomic measures such as residential history was another potential limitation of our study. We were unable to sufficiently estimate socioeconomic position using zip codes because a large percentage of patients in our region have postal office boxes. However, eastern North Carolina is largely homogenous with respect to socioeconomic status and it is unlikely that the inclusion of this information in our models would have substantively altered any study findings. Race was self-reported and there could have been potential misclassification of this variable. Additionally, genetic data were not collected and, therefore could not be accessed as a potential cofounder in our analyses. Chance or an unexplained paradox specific to our patient population also may underlie our findings.

The status of several variables in our analysis may have changed over time. We did not adjust for these variables in a time-dependent manner due to their potential to be in the causal pathway. Similarly, intraoperative and postoperative variables were not included in our multivariable models.

Cause of death is not recorded in the National Death Index and POAF status may have been unrelated to their mortality. Although we adjusted for known clinically relevant variables, we acknowledge that other unmeasured factors could have influenced our results due to the retrospective nature of this study.

The results of this study are from a rural region with a unique population and may not generalize to other regions of the country. However, because our data were collected from a single unified health system, this might have partially controlled for other healthcare-related factors.

## 4. Conclusions

Our study identified an independent preoperative risk factor for long-term mortality among blacks receiving CABG. This outcome provides information that may be useful for surgeons, primary care providers, and their patients. Further research, especially in the context of psychosocial and biological factors (as well as their interaction), is needed to validate our findings and to determine the potential explanation for the racial disparity observed in our study.
